# Exploring microbial dark matter to resolve the deep archaeal ancestry of eukaryotes

**DOI:** 10.1098/rstb.2014.0328

**Published:** 2015-09-26

**Authors:** Jimmy H. Saw, Anja Spang, Katarzyna Zaremba-Niedzwiedzka, Lina Juzokaite, Jeremy A. Dodsworth, Senthil K. Murugapiran, Dan R. Colman, Cristina Takacs-Vesbach, Brian P. Hedlund, Lionel Guy, Thijs J. G. Ettema

**Affiliations:** 1 Department of Cell and Molecular Biology, Science for Life Laboratory, Uppsala University, Uppsala, Sweden; 2 School of Life Sciences, University of Nevada Las Vegas, Las Vegas, NV, USA; 3 Department of Biology, University of New Mexico, Albuquerque, NM, USA; 4 Department of Medical Biochemistry and Microbiology, Uppsala University, Uppsala, Sweden

**Keywords:** Archaea, eukaryogenesis, metagenomics, microbial diversity, single-cell genomics, tree of life

## Abstract

The origin of eukaryotes represents an enigmatic puzzle, which is still lacking a number of essential pieces. Whereas it is currently accepted that the process of eukaryogenesis involved an interplay between a host cell and an alphaproteobacterial endosymbiont, we currently lack detailed information regarding the identity and nature of these players. A number of studies have provided increasing support for the emergence of the eukaryotic host cell from within the archaeal domain of life, displaying a specific affiliation with the archaeal TACK superphylum. Recent studies have shown that genomic exploration of yet-uncultivated archaea, the so-called archaeal ‘dark matter’, is able to provide unprecedented insights into the process of eukaryogenesis. Here, we provide an overview of state-of-the-art cultivation-independent approaches, and demonstrate how these methods were used to obtain draft genome sequences of several novel members of the TACK superphylum, including Lokiarchaeum, two representatives of the Miscellaneous Crenarchaeotal Group (Bathyarchaeota), and a *Korarchaeum*-related lineage. The maturation of cultivation-independent genomics approaches, as well as future developments in next-generation sequencing technologies, will revolutionize our current view of microbial evolution and diversity, and provide profound new insights into the early evolution of life, including the enigmatic origin of the eukaryotic cell.

## 1. Introduction

Eukaryogenesis represents a fundamental evolutionary transition in the history of life on Earth, and a better understanding of the underlying events is thus highly relevant. During the past decades, a plethora of hypotheses have been put forward to account for the evolution of the eukaryotic cell, but a consensus has not been reached so far (reviewed in references [1–7]). While it is widely accepted that mitochondria derived from a bacterium related to Alphaproteobacteria (reviewed in reference [8]), the identity of the host cell remains the subject of debate. In particular, recent discussions have focused on whether the progenitor of the eukaryotic cell was a bona fide archaeon related to the TACK superphylum (an archaeal clade originally comprising Thaumarchaeota, Aigarchaeota, Crenarchaeota and Korarchaeota) [9–13] or a protoeukaryotic cell which formed a sister relationship with the archaeal domain of life [14,15].

The recent discovery of the Lokiarchaeota [16], a deeply branching lineage of the TACK superphylum, has shed new light on these discussions. First, phylogenetic analyses of a conserved set of marker genes suggested that Lokiarchaeota form a monophyletic group that includes eukaryotes. Furthermore, the investigation of a lokiarchaeal composite genome revealed a plethora of eukaryotic signature proteins (ESPs) previously identified solely in eukaryotes including proteins involved in the ubiquitination pathway, ESCRT machinery components, cytoskeletal proteins such as actins, and a large number of small GTPases [16]. These findings not only lend further support to the emergence of eukaryotes from within the archaeal domain of life (consistent with a two domain topology [12]), but also illuminate some of the early steps leading to the evolution of important eukaryotic characteristics, such as those involved in the origin of the endomembrane system, cytoskeleton and phagocytosis. The discovery of Lokiarchaeota exemplifies that the generation of novel sequence data derived from yet-uncultivated archaeal lineages affiliating with the TACK superphylum will reveal a more detailed picture of the process of eukaryogenesis, and that it will help to obtain a better resolution of deep, domain-level evolutionary relationships [3,16]. Furthermore, genome analyses with a particular focus on the presence of ESPs will aid in the reconstruction of the evolutionary events that have been instrumental in the early stages of the origin of the eukaryotic cell [4,9,11,17].

**Figure 1.**
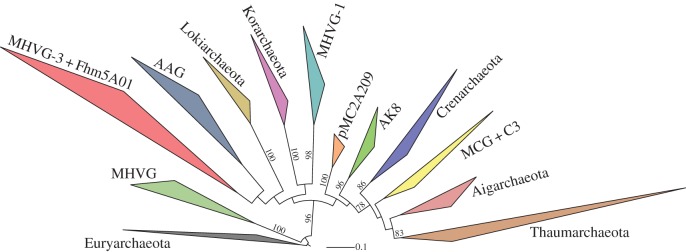
Phylogenetic diversity of the TACK superphylum based on known 16S rRNA gene sequences. Maximum-likelihood phylogeny of archaeal lineages within the TACK superphylum was constructed based on a total of 343 16S rRNA gene sequences. Five members of Euryarchaeota were used as outgroup to root the tree. Acronyms used for some of the archaeal clades are MHVG, marine hydrothermal vent group; AAG, ancient archaeal group. The TACK clades AK8, Fhm5A01 and pMC2A209 are derived from clone names. See figure S1 for a full, uncollapsed version of this tree.

Despite recent progress in cultivation-independent genomics approaches, many phylum- and order-level taxa still lack sequenced representatives; Lokiarchaeum is just one in a number of archaeal lineages affiliating with the TACK superphylum [9] (figure 1). Gathering genomic information for these lineages is challenging: many of them represent low-abundance community members and occur in hard-to-reach, little-explored environments including deep marine sediments and hydrothermal vent systems [18]. Clearly, ongoing efforts in the development of novel sequencing technologies and sequence analysis tools need to be pursued. In §2, we give an overview on the cultivation-independent biodiversity exploration approaches that exist and how they can be used to shed light on archaeal dark matter. In addition, we provide several examples of how these approaches were used to obtain genomic data of new TACK members, thereby revealing new insights into the dark ages of eukaryogenesis.

## 2. Metagenomic approaches for genomic exploration of microbial dark matter

**Figure 2.**
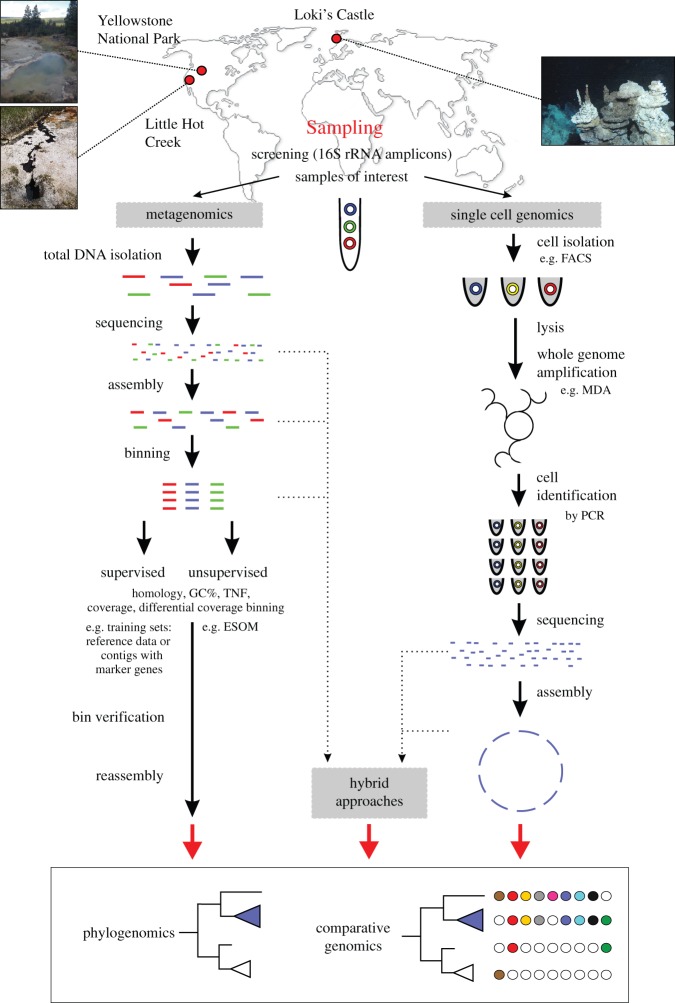
Overview of cultivation-independent approaches used to reconstruct microbial genomes. Schematic chart displays steps involved in cultivation-independent methods to characterize microbial dark matter. TNF, tetranucleotide frequency; ESOM, emergent self-organizing map; MDA, multiple-strand displacement amplification; FACS, fluorescent activated cell-sorting.

Metagenomics represents an important cultivation-independent approach to study microbial communities at the genomic level [19]. Since its conception in the early 2000s, the field of metagenomics has been revolutionized as a result of the development and maturation of high-throughput and massively parallel sequencing technologies. Currently, a typical metagenomic dataset comprises a large amount (up to billions) of short (paired-end) reads derived from entire microbial community DNA. High-quality assemblies yielding large contigs are, however, often difficult to achieve, likely owing to complex community structures, insufficient genome coverage and strain microdiversity. The main effort of obtaining genomic information from a single organism has therefore shifted towards *in silico* binning approaches that aim to group (‘bin’) contigs belonging to the same organism (figure 2). A variety of binning approaches have been developed and can broadly be divided into supervised and unsupervised methods.

Supervised tools require *a priori* information about the genome of interest as a basis for the extraction of additional contigs with similar sequence patterns. For instance, MEGAN [20] is based on homology searches, PhyloPythia/S/S+ [21–23] and ClaMS [24] compare sequence compositions, such as oligonucleotide frequencies, CARMA [25] performs phylogenetic reconstructions for sequence classification and PhymmBL [26,27] uses a combination of sequence composition and BLAST. Another hybrid approach, retrieving training sets from the metagenome itself, was recently also used to obtain a composite genome of the Lokiarchaeota [16].

As metagenomes often contain vast amounts of novel genomic data, the development of unsupervised methods that require no prior knowledge of the target genome is of major interest. These approaches rely solely on sequence composition characteristics, including GC content [28], tetranucleotide frequencies (TNFs) and related k-mer counting approaches (e.g. MetaCluster [29]). However, relying on such sequence characteristics alone has its limitations. For example, short contigs are prone to misclassification, and it is also intrinsically difficult to discriminate between contigs that originate from closely related strains. To overcome these limitations, read coverage information is often used in conjunction with sequence composition information (e.g. MaxBin [30] or MetaWatt [31]). A recent improvement in this realm of tools includes the differential coverage binning strategies, in which read coverage obtained by metagenomic sequencing closely related samples (e.g. from a time series, or different DNA extraction methods) is used to improve genomic binning [32]. Automatic clustering based on TNF and differential coverage is, for example, employed by CONCOCT [33] and GroopM [34]. Finally, another commonly used tool is based on emergent self-organizing maps in which clustering can not only be performed using TNFs [35], but also include read coverage information [36].

Regardless of the binning method used, careful inspection of the resulting ‘genome bins’ remains an absolute necessity. Standard quality assessments should include the identification of single copy marker genes and the verification that these are derived from a single taxonomic source. The presence of single copy marker genes can also be used for estimating bin completeness and redundancy.

## 3. Single-cell genomics as a tool for microbial dark matter exploration

Single-cell genomics (SCG) represents a powerful tool to complement metagenomics as it facilitates the genomic exploration of DNA from individual uncultured cells rather than from communities obtained by metagenomics (reviewed in references [37–39]). A commonly used approach to obtain single cells from environmental samples is based on fluorescence-activated cell sorting (FACS), whereas more recently developed cell sorting methods include microfluidics (e.g. [40,41] and reviewed in [42]) as well as optical tweezers [43,44] to capture individual cells (figure 2). Following sorting, individual cells are lysed, and the genomic DNA of each cell is amplified using multiple displacement amplification (MDA) or similar techniques, yielding single-cell amplified genomes (SAGs). SAGs of interest can be identified and selected for high-throughput sequencing using PCR-based screening (e.g. targeting marker genes such as 16S rRNA gene; figure 2).

While single cell and metagenomic approaches have revolutionized our insights into microbial dark matter [45,46], these methods still face some important challenges [47,48]. For example, metagenomics (particularly when applied to complex communities) requires the generation and assembly of large amounts of genomic data, which is computationally demanding. Furthermore, assemblers often have difficulties resolving strain-level microdiversity, which is a common feature of most natural microbial populations. Finally, despite ongoing efforts to improve binning methods, several problems have yet to be addressed including contamination from unrelated genomes with similar nucleotide frequencies. Additionally, binning can only recruit genomic fragments that have co-evolved with the originating genome, and thus recently acquired genes and phage or viral regions might not be part of the final assembly [39]. In SCG approaches, the necessity to pre-amplify genomic DNA causes artefacts such as uneven coverage and chimera formation during MDA reaction, often resulting in highly incomplete or fragmented genomes [39].

One possibility to improve the quality and completeness of genomic assemblies is to combine SCG and metagenomics. For instance, SAGs can be used to recruit fragments from metagenomes [49,50] or they can serve as training sets for supervised binning efforts of metagenomic data derived from the same sample (e.g. see §5). Reassembly of reads from a particular genome bin and corresponding SAG has the potential to improve the quality and completeness of the genome assembly and to obtain near-complete genomes (table 1).

**Table 1.** Summary of genome assembly statistics. Assembly statistics (number of contigs larger than 1 kbp, total length of contigs larger than 1 kbp, largest contig, G + C ratio of all contigs, N50, coding sequences (genes), and completeness) for the three SAGs and a metagenomic bin are shown.

	MCG SAG (10Y13-A3)	MCG SAG (10Y13-F10)	Korarchaeon SAG (LHC4)	Korarchaeon SAG (LHC4) [SAG + metagenome co-assembly]	Lokiarchaeum (metagenome bin)
contigs (>1 kbp)	139	73	117	140	504
total length (contigs >1 kbp)	819 884	717 176	1 228 747	1 488 773	5 143 417
largest contig (bp)	26 719	77 696	57 895	99 230	71 539
G + C ratio	37.3	31.26	47.3	47.4	31.3
N50	9355	26 221	25 432	23 488	15 403
CDS	779	746	1717	2074	5386
% completeness	38	50	87	89	92

In §4, we provide examples to clarify the principle of novel techniques applied in our research group to explore the genomic potential of novel TACK lineages using cultivation-independent approaches. So far, we have focused on generating single-cell and metagenomic sequence data from various sediment samples from hot springs and hydrothermal vent systems. These environments have previously been suggested to harbour a large diversity of so far uncultivated archaeal lineages [18,51], some of which may represent close relatives of the elusive ancestor of eukaryotes.

## 4. Single cell genomic identification of two novel Miscellaneous Crenarchaeotal Group-related lineages

**Figure 3.**
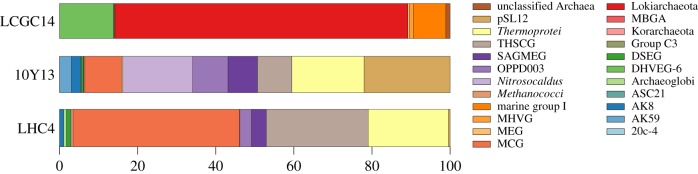
Archaeal diversity in environmental samples that were analysed. Stacked bar graphs showing abundance of different archaeal groups according to classification system used in Silva 16S rRNA database. Acronyms used for each archaeal group are shown next to the colour-coded legend. Total amount of archaeal OTUs identified from the samples were: 26 in LCGC14, 45 in 10Y13 and 33 in LHC4. Total archaeal abundances in the samples were: 11% for LCGC14, 11% for 10Y13 and 52% for LHC4.

Hot springs, for instance those located in Yellowstone National Park, represent hot spots of archaeal diversity [52]. Not only do hot springs host diverse archaeal model organisms [53], they often also contain a plethora of little-investigated archaeal lineages such as Korarchaeota [54,55] as well as a huge diversity of uncultivated archaeal lineages that are amenable to further study [56]. In this study, a previously uncharacterized hot spring in the Lower Culex region of the Lower Geyser Basin, Yellowstone National Park (GPS coordinates: 44°34′23.0″N 110°47′40.5″W) with temperatures around 70°C and pH of 8.6 was selected based on its high archaeal diversity. Screening of 16S rRNA gene amplicons generated from DNA isolated from sediments that were sampled during the spring of 2010 (sample 10Y13) showed high abundance of archaeal lineages from the TACK superphylum, such as the Miscellaneous Crenarchaeotal Group (MCG; 9%), *Nitrosocaldus* (18%), OPPD003 (9%), THSCG (9%), *Thermoprotei* (19%) and pSL12 (22%; figure 3). Therefore, this sample was selected to be analysed using single-cell genomics.

**Figure 4.**
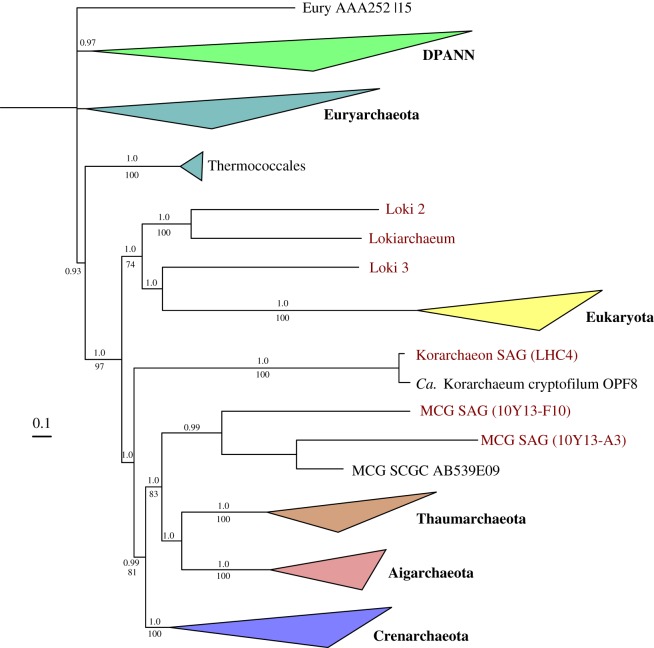
Placement of three newly sequenced SAGs and three metagenomic bins of novel uncultivated Archaea within the tree of life. Bayesian and maximum-likelihood analysis of major archaeal groups based on a concatenation of the alignment of 36 highly conserved marker genes present in all domains of life. Bayesian posterior probability (PP) values are shown on top and bootstrap (BS) values are shown below the branches. PP values less than 0.7 or BS values less than 70 are not shown. See electronic supplementary material, figure S2 for a full, uncollapsed version of this tree.

To extract cells from sediment sample 10Y13, a Nycodenz gradient centrifugation method was applied [49]. Cell fractions were then sorted by FACS into 384-well plates, followed by alkaline lysis and MDA to generate SAGs (see electronic supplementary material for details). A qPCR screen using Archaea-specific 16S rRNA gene primers identified 20 potential archaeal SAGs. Follow-up sequencing using an Illumina HiSeq instrument followed by genome assembly revealed that two of the SAGs, A3 and F10, belonged to uncultured archaeal lineages (see electronic supplementary material for details). Inspection of the genomic assemblies of these two SAGs, which had total assembly sizes of 0.8 and 0.7 Mbp for A3 and F10, respectively, indicated that these were lacking 16S rRNA genes, which hindered classical taxonomic classification. However, the extraction of single-copy marker genes from these SAGs allowed us to determine their phylogenetic affiliation. Both maximum-likelihood and Bayesian phylogenies using 36 concatenated marker genes placed the two SAGs in a clade with the archaeon belonging to MCG (also called ‘Bathyarchaeota’ [57]) from Lloyd *et al.* [58], indicating that the two SAGs represent novel thermophilic members, or distant relatives, of this candidate phylum (figure 4). Using single-copy marker genes, the completeness of these SAGs, referred to as MCG SAGs 10Y13-A3 and 10Y13-F10, was estimated to be about 38% and 50%, respectively. Analysis of the genomic content of these novel SAGs revealed some interesting eukaryotic features, including the presence of a ubiquitin protein modifier system, ESCRT-related proteins, topoisomerase IB and crenactin (see §5).

## 5. Metagenomic discovery of the Lokiarchaeota

In a recent microbial diversity survey of hydrothermal fields, marine sediments were sampled near Loki's Castle [59]. Analysis of a 16S rRNA gene amplicon library from these samples revealed that approximately 10% of the sequences (approx. 75% of the archaeal sequences) belonged to the Deep-Sea Archaeal Group/Marine Benthic Group B (referred to as DSAG here) [16] (figure 3), a clade that was previously hypothesized to be a deep-branching member of the TACK superphylum [9] (figure 1). To explore this clade at the genomic level, DNA was extracted from the sample and amplified, and 56 Gb of raw sequence data was assembled into 289 831 contigs larger than 1 kb [16].

To extract metagenomic contigs belonging to the DSAG clade, a supervised binning approach was developed that did not rely on the availability of reference genomes [16]. To constitute the necessary training sets, 59 robust taxonomic markers [3] were selected. All homologues of these markers were identified in the metagenomes, and single-gene trees of these markers complemented with about a hundred reference sequences were inferred. Trees were visually inspected, paying special attention to the placement of sequences found in the metagenomes. This allowed (i) the verification of the presence of the taxa that were inferred by 16S rRNA gene phylogenies, (ii) the estimation of microdiversity in each clade, and (iii) the identification of contigs that could be used as a training set for supervised metagenomic binning [26]. As noted in §2, microdiversity is known to complicate the analysis of metagenomes, as genome assemblers tend to assemble conserved regions of the genomes into collapsed, high-coverage contigs and more diverged regions into more numerous, low-coverage contigs. This is highly problematic for binning strategies relying on coverage, especially those using differential coverage [33]. However, in this project, this apparent problem was turned into an advantage: knowing the approximate number of closely related lineages of a particular archaeal clade significantly aided the identification of markers belonging to this group when marker gene trees were particularly difficult to interpret. Altogether, five archaeal clades could be defined in this way, resulting in the formation of five corresponding training sets (436 kb on average) that were subsequently used for supervised binning of metagenomic contigs. The bin corresponding to one of these clades (Lokiarchaeum) turned out to be highly abundant, and advanced coverage-based filtering and reassembly allowed for the reconstruction of a near-complete (92%) composite genome of 5.1 Mb (table 1), with a redundancy of 1.4.

**Figure 5.**
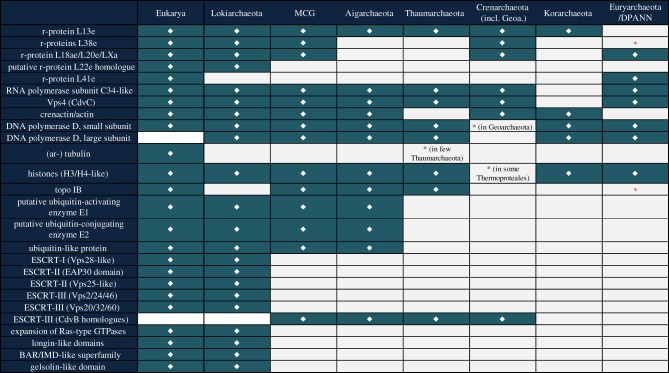
Overview of ESPs. Overview on distribution of ESPs in different archaeal phyla as well as in eukaryotes. An asterisk indicates cases where a respective ESP was found only in a subset of the lineages that comprise a given phylum.

Another bin (Loki2/3) turned out to contain two low-abundance TACK Archaea that were distantly related to Lokiarchaeum. Although complete genomes could not be recovered for these two, taxonomic markers present in the Loki2/3 bin could be separated based on their slight, but significant, difference in GC content (about 3%). After thorough phylogenomic analyses involving the 36 conserved marker genes mentioned above, the newly defined Lokiarchaeota phylum, comprising Lokiarchaeum, Loki2 and Loki3 turned out to be the closest archaeal relative to eukaryotes [16] (figure 4). The inferred common ancestry between Lokiarchaeota and eukaryotes was further reinforced by the presence of significant amounts of ESPs in the Lokiarchaeum genome (see below, and figure 5), which suggests that the archaeal ancestor of eukaryotes might have had a relatively complex membrane biology that included primitive vesicle formation and trafficking capabilities [16].

## 6. A new korarchaeal genome obtained by combining single-cell and metagenomic data

Combining single-cell and metagenomic datasets has the potential to recover near-complete microbial genomes. Here, we used this approach to obtain a near-complete genome of a member of the Korarchaeota by combining an NGS dataset from a single korarchaeal cell and a metagenomic dataset retrieved from a hot spring sediment sample in Little Hot Creek (LHC; CA, USA). The LHC sample comprises considerable diversity of Archaea and Bacteria but only one Korarchaeota phylotype was identified from amplicon data (figure 3). The strategy was to recruit or recover raw korarchaeal sequence reads from the metagenome to supplement the single-cell genome data, and to improve assembly quality and completeness (see electronic supplementary material for details).

Using the contigs of the LHC Korarchaeon SAG as one of the training datasets, contigs larger than 1 kbp from the LHC metagenome were taxonomically classified using PhymmBL [26] and reads assigned to Korarchaeota were retrieved (see the electronic supplementary material for details). Where traditional read recruitment methods using BLAST or read aligner would fail to recover missing genomic regions in the SAG assembly, the use of the PhymmBL allowed identification of contigs (and hence reads) belonging to the LHC Korarchaeon, which were not present in the SAG assembly. By co-assembling the retrieved metagenomic reads with those from the SAG data, an improved assembly was achieved (table 1). Co-assembly of the SAG and metagenomic reads increased the total assembly size (by over 260 kbp) as well as the largest contig (by more than 40 kbp). In addition, the estimated completeness of the co-assembled genome (1.48 Mbp) was 89%, an improvement of 2% (table 1). It is also notable that no contaminating marker genes were identified in the co-assembly, an indication of its high quality.

A comparison of the partial 16S rRNA gene recovered from the LHC Korarchaeon with that of the sequenced ‘*Candidatus* Korarchaeum cryptofilum’ OPF8 strain indicated that these are 97% identical. In addition, both sequences form a highly supported clade in 16S rDNA gene-based maximum-likelihood phylogenies, supporting their close affiliation (data not shown). Using a set of 36 single-copy marker genes known to be present in all domains of life, Bayesian and maximum-likelihood phylogenies were constructed that placed the expanded korarchaeal clade at the base of a clade comprising Thaumarchaeota, Aigarchaeota, Crenarchaeota and MCG with significant bootstrap and posterior probability support values (figure 4).

## 7. Eukaryotic signature proteins in novel TACK Archaea provide new insights into eukaryogenesis

Eukaryotic genomes encode proteins of mixed phylogenetic heritage. For instance, many eukaryotic proteins involved in central metabolism and membrane chemistry show similarity to bacterial proteins and a subset of these have been inferred to originate from the bacterial endosymbiont from which mitochondria evolved [60–66]. In contrast, eukaryotes and Archaea share core subunits of several informational processing machineries, including ribosomal, transcriptional and replicative complexes [4,64,67,68]. During recent years, comparative genomic analyses have revealed that lineages affiliating with the archaeal TACK superphylum share an additional subset of ESPs with eukaryotes [9]. For instance, genomes of Thaumarchaeota, an archaeal phylum comprising ammonia-oxidizing archaea, revealed the presence of topoisomerase IB, which formed a sister relationship with eukaryotic homologues in phylogenetic analyses [69]. In addition, a novel cell division system was described to function in some Crenarchaeota, which involved distant homologues of eukaryotic ESCRT-III proteins as well as an ATPase related to vacuolar protein sorting-associated protein 4 [70–72]. Additional ESPs found in members of the TACK superphylum include distant archaeal homologues of eukaryotic actins referred to as crenactins [17,73,74] and tubulins, denoted ar-tubulins [75]. In addition, an additional DNA-dependent RNA polymerase subunit, Rpb8, was identified in Korarchaeota and Crenarchaeota [76], and a ubiquitin-like protein modifier system in ‘*Candidatus* Caldiarchaeum subterraneum’ [77], the first representative of Aigarchaeota (figure 5).

The current expansion of deeply branching members of the TACK superphylum, including two MCG-related lineages, one novel member of the Korarchaeota and Lokiarchaeum, has significantly expanded this set of ESPs in archaeal genomes. Notably, the MCG-like SAGs A3 and F10, which are thermophilic representatives of the abundant and widespread MCGs [78], unite all archaeal cell division proteins including crenactin, CdvABC and FtsZ homologues. Additional ESPs, previously identified in just a subset of other archaeal lineages (figure 5) and including for instance histones, topoisomerase IB, ubiquitin modifier system-related proteins and ribosomal protein L38, were also encoded by these SAGs.

**Figure 6.**
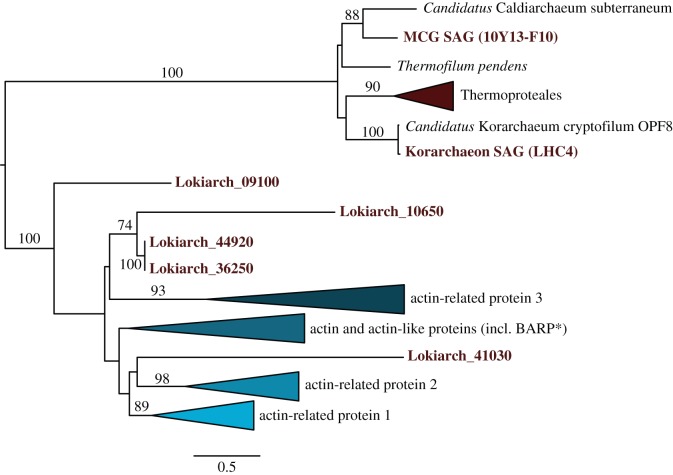
Actin phylogeny. Maximum-likelihood phylogeny of 378 aligned amino acid residues of eukaryotic actins, Arp1-3 homologues and crenactin including homologues identified in Lokiarchaeum as well as the MCG SAG (10Y13-F10) and Korarchaeon SAG (LHC4). Only bootstrap values larger than 70 are shown. BARP, bacterial actin-related protein.

However, even more surprising was the finding of a large amount of additional ESPs in the first genome of a member of the Lokiarchaeota [16] (figure 5). First of all, Lokiarchaeum is the first archaeon that encodes homologues of bona fide eukaryotic actins and related actin-like proteins (figure 6). Phylogenetic analyses of archaeal and eukaryotic actins revealed that ‘Lokiactins’ are more closely related to eukaryotic actins than to actin homologues of other archaeal lineages including MCG SAG F10 (figure 6). Surprisingly, Lokiarchaeum was also found to encode small gelsolin-domain containing proteins, which in eukaryotes are part of actin binding, capping and modulating proteins, thus representing important factors in the regulation of actin cytoskeleton dynamics. Actin binding proteins have likely evolved from small gelsolin-like domains [79], which, given their presence in Lokiarchaeum, can be inferred to have been present in the last archaeal ancestor of eukaryotes.

Furthermore, the Lokiarchaeum genome revealed proteins with homology to components of eukaryotic multivesicular endosomal complexes ESCRT-III as well as to ESCRT-II and ESCRT-I [16]. The latter two of these complexes have so far been assumed to be restricted to eukaryotes and have not previously been identified in archaeal genomes. For instance, Lokiarchaeum encodes both an EAP30-domain containing protein and a Vps25 homologue, both of which are part of ESCRT-II in eukaryotes. Additionally, a putative Vps28 homologue, which is a component of ESCRT-I, was found. Interestingly, it also encodes two different types of SNF7 domain proteins (part of ESCRT-III), which appear to share common ancestry with each of the eukaryotic Vps2/24/46 and Vps20/32/60 subfamilies [16], respectively, rather than being closely related to archaeal SNF7 family proteins. Altogether, these findings suggest that several important building blocks of the endosomal sorting complexes originated in Archaea. The presence of the ubiquitin protein modifier system in the Lokiarchaeum genome opens up the possibility that, similar to eukaryotes, Lokiarchaeum has the ability to degrade ubiquitinated target proteins via a primitive ESCRT pathway.

Surprisingly, the Lokiarchaeum genome revealed an unprecedented expansion of ‘eukaryotic’ small GTPase homologues of the Ras- and Arf-superfamilies, previously assumed to be a unique feature of eukaryotic genomes. The relative amount of genes encoding small GTPases in the Lokiarchaeum is comparable to that of eukaryotes, in which they function in a multitude of regulatory processes related to cytoskeleton remodelling, signal-transduction and vesicular trafficking [16]. The function of these ‘molecular switches’ in Lokiarchaeum is elusive so far, and it remains to be shown whether some of the various GTPase subgroups present in Lokiarchaeum represent direct relatives of eukaryotic families.

Finally, it is interesting to note that the Lokiarchaeum genome appears to encode the most eukaryotic-like ribosome so far, including a putative homologue of eukaryotic-specific r-protein L22e, in addition to all other ribosomal proteins hitherto identified in only a subset of archaeal lineages.

Intriguingly, many of the ESPs that have now been identified in Lokiarchaeum and other TACK members are components of various membrane remodelling activities in eukaryotes, including the formation and trafficking of vesicles and cell shape formation processes such as phagocytosis. The unification of many of these ESPs in Lokiarchaeota, which comprise the closest known relatives of eukaryotes to date, strongly suggests that a certain level of cellular complexity has originated in Archaea and preceded the endosymbiotic event that gave rise to mitochondria [16]. However, given the vast majority of as yet unknown archaeal lineages, some of which will have perhaps even closer phylogenetic affiliation to eukaryotes, it is likely that Lokiarchaeum has only revealed a glimpse of the evolutionary steps that led to the origin of the eukaryotic cell. Prospective genomic analyses will certainly unravel additional insights into the emergence of cellular complexity in eukaryotes. Moreover, cell biological studies of these organisms will aid in shedding more light onto the function of these ESPs in an archaeal context.

## 8. Concluding remarks

The maturation of next-generation sequencing technologies and the development of a multitude of cultivation-independent approaches have resulted in a plethora of genome sequence data of hitherto uncultivated microorganisms. The results of several exploratory ‘dark matter’ projects have already forced us to rethink our tenets regarding the diversity, ecology and evolution of the microbial world. With respect to the theme of the current contribution, the recent genomic discovery and exploration of new lineages affiliated with the archaeal TACK superphylum, such as the Lokiarchaeota, has revolutionized the field of eukaryogenesis. The future development of new and more powerful sequencing technologies, combined with the development of bioinformatics tools that allow for the reconstruction of near-complete genomes, will continue to revolutionize microbial dark matter exploration. Undoubtedly, the future genomic exploration of novel TACK-related Archaea will provide more details about the identity and nature of the elusive archaeal ancestor of eukaryotes.

## Supplementary Material

Supplementary Material

## Data accessibility

The datasets supporting this article have been uploaded as part of the electronic supplementary material and deposited in Dryad: http://datadryad.org/review?doi=doi:10.5061/dryad.df264.
