# Supplementary material for: Exploring microbial dark matter to resolve the deep archaeal ancestry of eukaryotes
Source: Philos Trans R Soc Lond B Biol Sci. 2015 Sep 26;370(1678):20140328. doi: 10.1098/rstb.2014.0328 (PMC4571567; doi:10.1098/rstb.2014.0328)
Supplement: Supplementary Material [file rstb20140328supp1.pdf]

Supplementary material for Saw *et al*, 2015, Exploring microbial dark matter to resolve the deep archaeal ancestry of eukaryotes, *Phil. Trans. R. Soc. B.* doi: 10.1098/rstb.2014.0328

# Exploring microbial dark matter to resolve the deep archaeal ancestry of eukaryotes

## Sampling sites

Environmental samples discussed in this study were collected from three locations. Hot spring sediment sample 10Y13 was collected from a hot spring near Lower Culex Basin (44°34'23.0"N 110°47'40.5"W) in Yellowstone National Park (YNP), USA. The temperature of the hot spring from which the sample was taken was 68.8°C with pH of 8.6. Hot spring sediments appeared light brown in colour. Deep-sea sediment sample LCGC14 was subsampled at 75 cm below sea floor from a 2-meter-long gravity core retrieved from the Arctic Mid-Ocean Spreading Ridge (73°45'47.4"N 8°27'50.4"W) at a depth of 3283 meters below sea level [1]. LHC4 sample was taken from a hot spring located in the Long Valley Caldera, near Mammoth Lakes, CA, USA (37°41'26.2"N 118°50'39.2"W). The temperature of this hot spring was around 80°C with near-neutral pH and black sediment [2].

## Amplicon data

Amplification and sequencing of the V4 region of 16S rRNA genes of Bacteria and Archaea from LHC4 sample was performed as described in Kozich et al [3] with the following modifications: to be more inclusive of several archaeal lineages, forward primer 515F was modified to contain a C or T at the 4th position from the 5' end (5'-GTGYCAGCMGCCGCGGTAA-3'), and a corresponding modification was made to the read 1 sequencing primer. PCR was conducted using 5 Prime HotMasterMix DNA polymerase (#2200410, 5 Prime Inc., Gaithersburg, MD, USA) and included 33 amplification cycles. Amplicons were sequenced on the Illumina MiSeq platform at Micro-Seq Enterprises (Las Vegas, NV, USA).

For Loki's Castle sediment and Yellowstone hot spring sediment samples, 'universal' A519F (5'-CAGCMGCCGCGGTAA-3') and U1391R (5'-ACGGGCGGTGWGTRC-3') primers were used to amplify ~900 bp fragment of the 16S rRNA gene spanning V3 to V8 regions. Detailed methods for PCR conditions and library construction are described in a previous study [1]. Amplicons were generated with Illumina MiSeq instrument.

© The Authors under the terms of the Creative Commons Attribution License http://creativecommons.org/licenses/by/3.0/, which permits unrestricted use, provided the original author and source are credited.

A total of 118 Mbp, 506 Mbp, and 21 Mbp of raw Illumina MiSeq amplicon data were generated for Loki's Castle, Yellowstone, and LHC4 samples, respectively. The 5' and 3' regions of the amplicons were extracted by scanning for respective forward and reverse primers in read pairs using a custom Python script. Amplicon reads with average Phred quality below 30 were discarded from the analysis. As the amplicon size was roughly 900 bp, the read pairs could not be merged and were analyzed separately. In this analysis, only the 5' end of the amplicon extracted from both read pairs was used for estimation of OTU and phylum-level diversity. UPARSE pipeline [4] was used for further quality filtering, clustering of OTUs and chimera filtering. Resulting OTUs were classified by searching against Silva 16S rRNA data (release 119) [5] using BLASTn [6] with maximum E-value cutoff of $1e^{-5}$ and minimum identity threshold of 85%.

**Analysis of SAG data**

SAG data from YNP was generated by first obtaining cell fractions from hot spring sediment samples using Nycodenz gradient centrifugation, FACS for sorting individual cells into 384-well microtiter plates, alkaline lysis and multiple displacement amplification (MDA) at the Bigelow Single Cell Genomic Center. Sequencing libraries and reads were generated by the SNP&SEQ sequencing facility at Uppsala University. Total raw Illumina HiSeq data (paired-end 2x100 bp) generated for the two SAGs ranged from 1.5 Gbp for MCG SAG (10Y13-A3) to 2.0 Gbp for MCG SAG (10Y13-F10).

Detailed methods for single-cell isolation and generation of SAG data for the Korarchaeon from LHC4 sample has previously been described in a study by Dodsworth et al [7]. One microliter of the original SAG DNA of the Korarchaeon was used to perform another MDA reaction to generate additional SAG DNA for library preparation. MDA reaction was performed using Qiagen REPLI-g Mini kit and purified with QIAamp DNA Mini kit (Qiagen, Hilden, Germany). A sequencing library was generated from 1 ng of the resulting SAG DNA with NexteraXT library preparation kit (Illumina, San Diego, CA, USA) and the library was sequenced with Illumina MiSeq instrument. Total raw Illumina MiSeq data (2x300 bp) generated for the Korarchaeon SAG was 4.9 Gbp.

Fastqc tool (http://www.bioinformatics.babraham.ac.uk/projects/fastqc/) was used to assess sequence quality and Illumina adapters were trimmed using Scythe (https://github.com/vsbuffalo/scythe). The qualities of adapter-trimmed reads were further improved using Sickle (https://github.com/najoshi/sickle) to only keep reads with Phred quality scores higher

than 30. Resulting trimmed and quality-filtered reads were assembled with SPAdes version 3.1.1 [8] using the flags "--sc" and "--careful", as well as default k-mers, i.e. 21, 33, and 55 for the HiSeq data and 21, 33, 55, 75, 101, and 125 for the MiSeq data.

**Generation of metagenomic data**

DNA was extracted from sediment from LHC4, collected on 01 May 2011, using the FastDNA Spin Kit for Soil (MP Biomedicals, Solon, Ohio, USA) and quantified by NanoDrop spectrophotometer (Thermo Scientific). Libraries were constructed using the Nextera DNA Sample Preparation Kit (Illumina). Two separate sequencing libraries were generated from the LHC4 sample: a MiSeq library with a total of 7.3 Gbp and a HiSeq library with a total of 10.8 Gbp of raw sequence data. Sickle was used to remove completely or trim low quality sequence ends with Phred quality score below 30. Quality-trimmed sequences (5.7 Gbp for MiSeq and 10.0 Gbp for HiSeq) from both libraries were assembled using Ray Meta version 2.3.1 [9] with k-mer of 35 and utilizing 1280 CPU cores on Beskow supercomputing cluster at PDC Center for High Performance Computing at KTH (Kungliga Tekniska Högskolan, Sweden).

**Generation of Loki's Castle metagenome**

Loki's Castle metagenomic reads were generated from sediment samples collected via gravity coring method [1]. Briefly, community DNA from the 7.5 g of raw sediment materials was extracted using FastDNA spin kit and quantified using fluorescent nanodrop ND-3300 instrument (Thermo Scientific) to measure dsDNA concentration. Sequencing library for Illumina HiSeq instrument was then generated using Nextera library preparation kit from Illumina. Amount of raw data generated by the HiSeq 2500 instrument was about 13.1 Gbp. Detailed assembly and further downstream analysis of the Loki's Castle metagenomic data was described in Spang et al [1].

**Binning of metagenomic contigs using PhymmBL**

Contigs belonging to target organisms were identified using supervised binning with PhymmBL [10], which was trained with available reference genomes (for instance clean contigs from Korarchaeon SAG was used to train PhymmBL). Additional manually added training sets included manually curated, contamination-free SAG assemblies. A training set comprising contigs of Lokiarchaeota

present in Loki Castle sediment samples was generated using the following approach: Phylogenetic trees of individual taxonomic marker genes were constructed and manually inspected. Comparison with the archaeal species tree allowed identification of contigs belonging to Lokiarchaeota (e.g. placement of genes from metagenomic contigs as deep-branching members of TACK superphylum indicated that these originated from Lokiarchaeota). Following several rounds of verification of this training set (see Spang et al for details [1]) these contigs were then used for supervised binning using PhymmBL. Only contigs larger than 1 kbp were analysed.

**Co-assembly of new Korarcheota**

A total of 15.2 million quality-trimmed and adapter-removed Illumina MiSeq reads from the SAG data and 237974 Illumina HiSeq reads mapped against contigs classified as Korarcheota by PhymmBL were assembled with SPAdes [8] using a set of k-mer sizes (21,33,55,75,101,125) and the "--sc" and "--careful" flags to handle single-cell data.

**Estimation of genome completeness**

Using a set of 162 marker genes known to be present in single copies in most archaea [11], genome completeness and redundancy was estimated by counting these marker genes in the three SAGs and three metagenomic bins in this study. This was accomplished by running PSI-BLAST of alignment profiles of marker genes from a representative set of archaea including all major lineages against the proteomes of these SAGs or metagenomic bins.

**Phylogenetic analyses**

**Phylogeny of 16S rRNA genes.** A total of 338 16S rRNA gene sequences representing major taxonomic clades from the TACK superphylum and members of deeply branching archaea from Silva release 119 were selected. Five taxa from Euryarchaeota were chosen as outgroup clade. Sequences were aligned with MAFFT L-INS-i [12] and columns with gaps if present in >50% of the taxa were removed with trimAl tool [13]. Maximum likelihood phylogeny was constructed using RAxML version 8.0.22 [14], using GTRGAMMA model of nucleotide substitutions and 100 bootstrap replicates.

**Phylogenetic analysis of concatenated marker proteins**. A set of 36 conserved single-copy marker genes were identified in the SAGs using PSI-BLAST and aligned with those from a select group of archaea, bacteria, and eukaryotes using the MAFFT L-INS-i tool. Alignments were trimmed using trimAl to remove column positions containing gaps in more than 50% of the taxa and subsequently concatenated. Maximum likelihood phylogeny was inferred using RAxML with 100 bootstrap replicates. Bayesian phylogenetic analyses were performed using Phylobayes [15], utilizing CAT+GTR model of amino acid substitution and 4 independent chains for ~10,000 generations. Consensus tree was generated with bpcomp tool from Phylobayes package to discard the first 2000 generations from the chains and sampling trees once every 50 generations. Bootstrap values from RAxML maximum likelihood tree were mapped on to corresponding positions in the Phylobayes consensus tree using "sumtrees.py" tool from DendroPy package [16].

**Actin phylogeny**. Representatives for major eukaryotic actin families (actins and ARP 1-3) [17, 18] as well as for crenactins (arCOG05583) were retrieved from NCBI and merged with actin homologs identified in Lokiarchaeum [1] as well as in the archaeal SAGs obtained in this study. The selected sequences were aligned using MAFFT L-INS-i and trimmed with trimAl to retain only those columns present in at least 50% of the sequences. Alignments were subjected to Maximum likelihood phylogenetic analyses using RAxML (8.0.22, GAMMA-LG) with the slow bootstrap option (100 bootstraps).

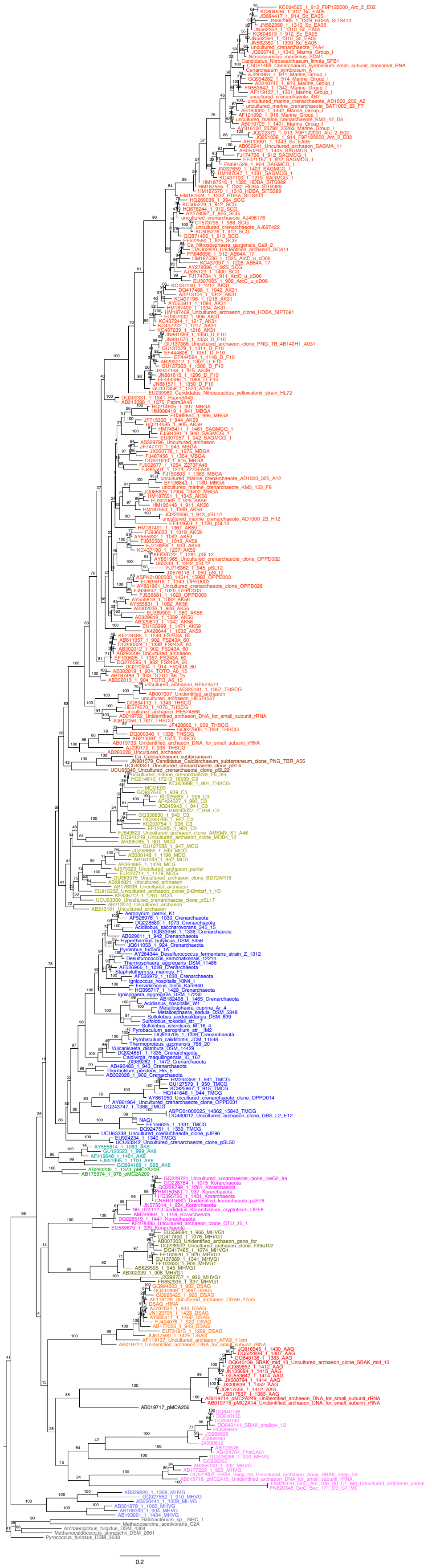

**Supplementary Figure 1:** Maximum likelihood phylogenetic tree of archaeal lineages within the TACK superphylum showing all major clades as classified in the Silva rRNA gene database.

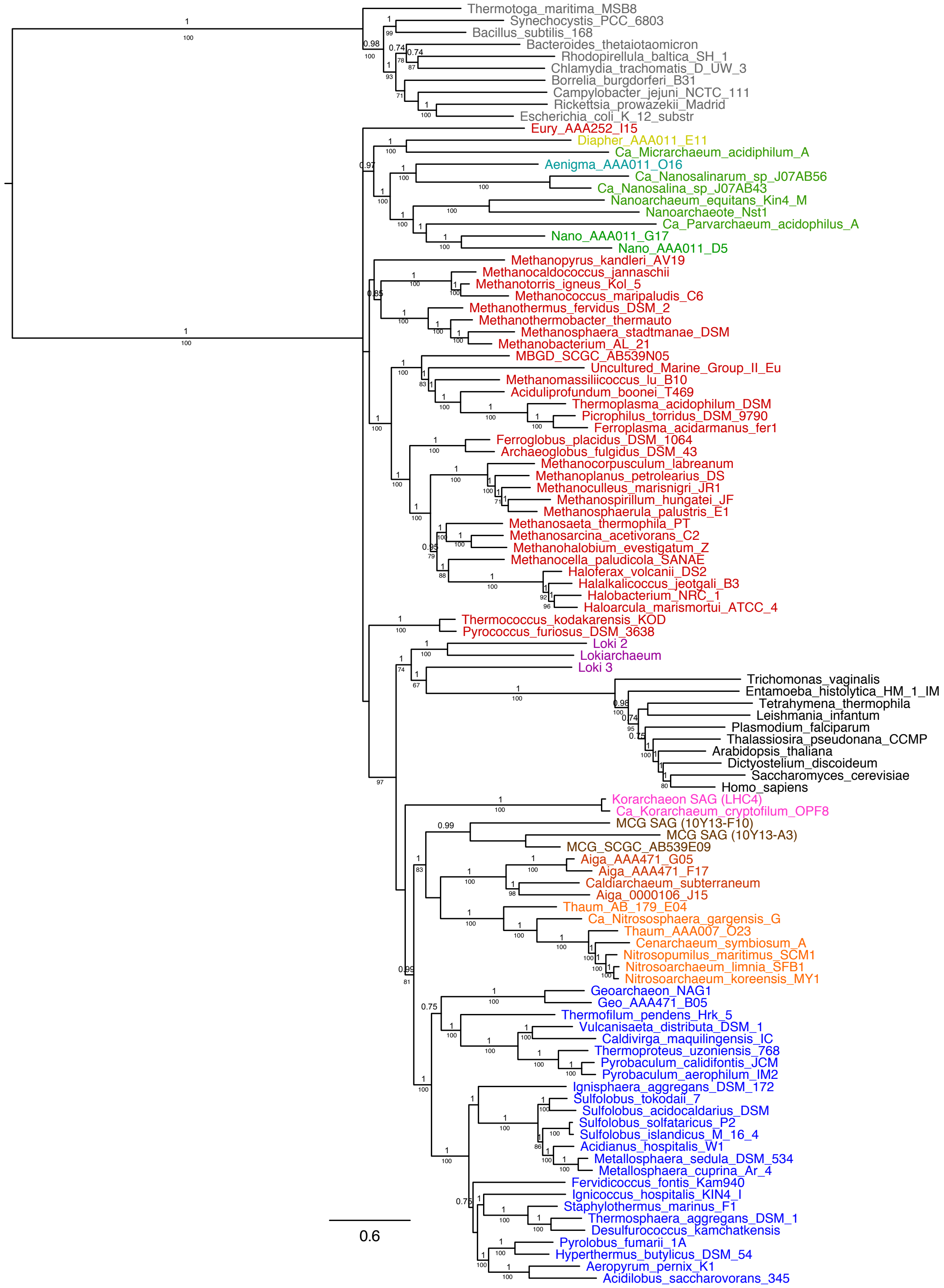


**Supplementary Figure 2:** Combined maximum likelihood and Bayesian phylogenetic tree showing all taxa. Bootstrap values were mapped onto the backbone tree produced by Phylobayes using “sumtrees.py” tool.

## Supplementary References


1. Spang, A., Saw, J.H., Jørgensen, S.L., Zaremba-Niedzwiedzka, K., Martijn, J., Lind, A.E., van Eijk, R., Schleper, C., Guy, L. & Ettema, T.J.G. 2015 Complex archaea that bridge the gap between prokaryotes and eukaryotes. **In press**.
2. Vick, T.J., Dodsworth, J.A., Costa, K.C., Shock, E.L. & Hedlund, B.P. 2010 Microbiology and geochemistry of Little Hot Creek, a hot spring environment in the Long Valley Caldera. *Geobiology* **8**, 140-154. (doi:10.1111/j.1472-4669.2009.00228.x).
3. Kozich, J.J., Westcott, S.L., Baxter, N.T., Highlander, S.K. & Schloss, P.D. 2013 Development of a dual-index sequencing strategy and curation pipeline for analyzing amplicon sequence data on the MiSeq Illumina sequencing platform. *Applied and environmental microbiology* **79**, 5112-5120. (doi:10.1128/AEM.01043-13).
4. Edgar, R.C. 2013 UPARSE: highly accurate OTU sequences from microbial amplicon reads. *Nature methods* **10**, 996-998. (doi:10.1038/nmeth.2604).
5. Yilmaz, P., Parfrey, L.W., Yarza, P., Gerken, J., Pruesse, E., Quast, C., Schweer, T., Peplies, J., Ludwig, W. & Glockner, F.O. 2014 The SILVA and "All-species Living Tree Project (LTP)" taxonomic frameworks. *Nucleic acids research* **42**, D643-648. (doi:10.1093/nar/gkt1209).
6. Altschul, S.F., Madden, T.L., Schaffer, A.A., Zhang, J., Zhang, Z., Miller, W. & Lipman, D.J. 1997 Gapped BLAST and PSI-BLAST: a new generation of protein database search programs. *Nucleic acids research* **25**, 3389-3402.
7. Dodsworth, J.A., Blainey, P.C., Murugapiran, S.K., Swingley, W.D., Ross, C.A., Tringe, S.G., Chain, P.S., Scholz, M.B., Lo, C.C., Raymond, J., et al. 2013 Single-cell and metagenomic analyses indicate a fermentative and saccharolytic lifestyle for members of the OP9 lineage. *Nature communications* **4**, 1854. (doi:10.1038/ncomms2884).
8. Bankevich, A., Nurk, S., Antipov, D., Gurevich, A.A., Dvorkin, M., Kulikov, A.S., Lesin, V.M., Nikolenko, S.I., Pham, S., Prjibelski, A.D., et al. 2012 SPAdes: a new genome assembly algorithm and its applications to single-cell sequencing. *Journal of computational biology : a journal of computational molecular cell biology* **19**, 455-477. (doi:10.1089/cmb.2012.0021).
9. Boisvert, S., Raymond, F., Godzaridis, E., Laviolette, F. & Corbeil, J. 2012 Ray Meta: scalable de novo metagenome assembly and profiling. *Genome biology* **13**, R122. (doi:10.1186/gb-2012-13-12-r122).
10. Brady, A. & Salzberg, S.L. 2009 Phymm and PhymmBL: metagenomic phylogenetic classification with interpolated Markov models. *Nature methods* **6**, 673-676. (doi:10.1038/nmeth.1358).
11. Rinke, C., Schwientek, P., Sczyrba, A., Ivanova, N.N., Anderson, I.J., Cheng, J.F., Darling, A., Malfatti, S., Swan, B.K., Gies, E.A., et al. 2013 Insights into the phylogeny and coding potential of microbial dark matter. *Nature* **499**, 431-437. (doi:10.1038/nature12352).
12. Katoh, K., Misawa, K., Kuma, K. & Miyata, T. 2002 MAFFT: a novel method for rapid multiple sequence alignment based on fast Fourier transform. *Nucleic acids research* **30**, 3059-3066.
13. Capella-Gutierrez, S., Silla-Martinez, J.M. & Gabaldon, T. 2009 trimAl: a tool for automated alignment trimming in large-scale phylogenetic analyses. *Bioinformatics* **25**, 1972-1973. (doi:10.1093/bioinformatics/btp348).
14. Stamatakis, A. 2014 RAxML version 8: a tool for phylogenetic analysis and post-analysis of large phylogenies. *Bioinformatics* **30**, 1312-1313. (doi:10.1093/bioinformatics/btu033).
15. Lartillot, N., Rodrigue, N., Stubbs, D. & Richer, J. 2013 PhyloBayes MPI: phylogenetic reconstruction with infinite mixtures of profiles in a parallel environment. *Systematic biology* **62**, 611-615. (doi:10.1093/sysbio/syt022).
16. Sukumaran, J. & Holder, M.T. 2010 DendroPy: a Python library for phylogenetic computing. *Bioinformatics* **26**, 1569-1571. (doi:10.1093/bioinformatics/btq228).
17. Goodson, H.V. & Hawse, W.F. 2002 Molecular evolution of the actin family. *Journal of cell science* **115**, 2619-2622.
18. Yutin, N., Wolf, M.Y., Wolf, Y.I. & Koonin, E.V. 2009 The origins of phagocytosis and eukaryogenesis. *Biology direct* **4**, 9. (doi:10.1186/1745-6150-4-9).
